# Supplementary material for: Identification of photoperiod-induced specific miRNAs in the adrenal glands of Sunite sheep (Ovis aries)
Source: Front Vet Sci. 2022 Jul 22;9:888207. doi: 10.3389/fvets.2022.888207 (PMC9354845; doi:10.3389/fvets.2022.888207)
Supplement: Supplementary file 1 [file Data_Sheet_1.ZIP › Supplementary file/miRDeep2 file.zip!/miRDeep2 file/Novel_Structure/SPLP42A2/Novel_545.pdf]

Provisional ID	:	NC_019470.2_26439
Score total	:	8.6
Score for star read(s)	:	3.9
Score for read counts	:	1.7
Score for mfe	:	1.4
Score for randfold	:	1.6
Score for cons. seed	:	
Total read count	:	15
Mature read count	:	9
Loop read count	:	0
Star read count	:	6

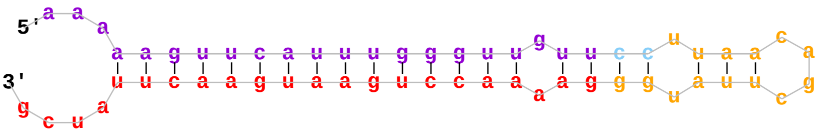

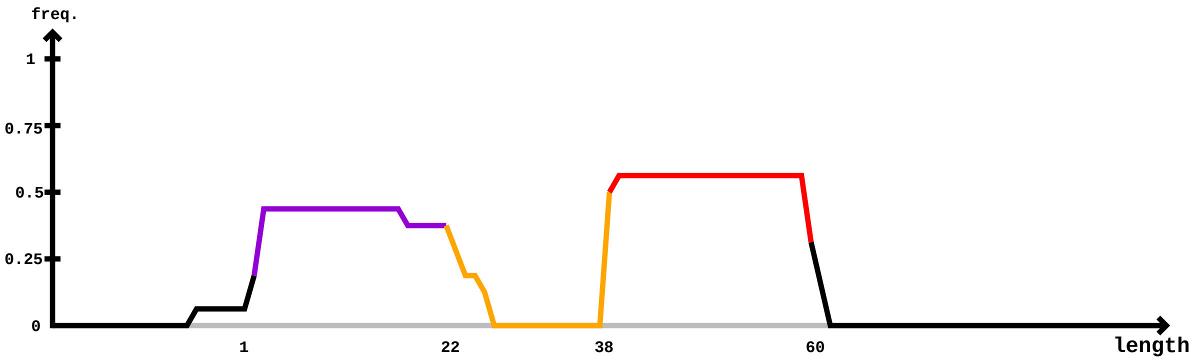

Star Mature

```
5'- uacuacuauuguguuggccaaaaaguucauuuggguuguuccuuaacagcuuauggaaaaccugaaugaacuuaucggucaacccaaaacguuagagaaauguagagugacag   -3'    obs
    uacuacuauuguguuggccaaaaaguucauuuggguuguuccuuaacagcuuauggaaaaccugaaugaacuuaucggucaacccaaaacguuagagaaauguagagugacag          exp
    ((((.((((((.(((((((...((((((((((((((.((((.(((....))).)))).)))))))))))))))...))))))).))..............))))))))....   reads      mm         sample
    .............uuggGcaaaaaguucauuuggg...............................................................................   1          1          seq
    ...................aaaaaguucauuuggguuguu..........................................................................   2          0          seq
    ....................aaaaguucauuuggguuguuG.........................................................................   1          1          seq
    ....................aaaaguucauuuggguuguuccA.......................................................................   1          1          seq
    ....................aaaaguucauuuggguuguuccuu......................................................................   2          0          seq
    ........................................................gaaaaccugaaugaacuuauc.....................................   4          0          seq
    ........................................................gaaaaccugaaugaacuuaucg....................................   4          0          seq
    .........................................................aaaaccugaaugaacuuaucg....................................   1          0          seq
```
